# Dark side of the shoulder: suprascapular and axillary nerve compressions

**DOI:** 10.1007/s00264-025-06465-9

**Published:** 2025-03-14

**Authors:** Gokhan Ayik, Ulas Can Kolac, Mehmet Kaymakoglu, Edward McFarland, Gazi Huri

**Affiliations:** 1 Yuksek Ihtisas Universitesi, Ankara, Turkey; 2 Hacettepe University, Ankara, Turkey; 3 Izmir University of Economics, Izmir, Turkey; 4 Johns Hopkins University, Baltimore, USA; 5 Aspetar FIFA Center of Excellence Orthopaedcis and Sports Medicine Hospital, Doha, Qatar

**Keywords:** Suprascapular nerve entrapment, Axillary nerve entrapment, Shoulder, Nerve, Weakness, Muscle atrophy

## Abstract

**Background:**

The suprascapular and axillary nerves can be subject to entrapment due to both their anatomical courses and their anatomical relationships with surrounding anatomical structures around shoulder. These entrapments were previously considered as a diagnosis of exclusion. However, today these pathologies can be diagnosed as primary. The most common complaints of patients are pain and sometimes weakness. The clinician’s suspicion is very important in making diagnosis. The patient’s history, duration of symptoms, and information such as the movements in which the complaints increase should be questioned carefully and in detail. In physical examination, symmetrical evaluation of both shoulders can provide important information. In addition, cervical and brachial plexus pathologies should be kept in mind. According to the suprascapular and axillary nerve innervations, muscle atrophy should be evaluated during inspection. Range of motion and neurological examination around shoulder should be performed. Since these entrapments can be seen together with rotator cuff tears and labrum pathologies etc., these additional pathologies should also be targeted during evaluation. The evaluation should be expanded with imaging methods such as plain radiographs, ultrasonography, computed tomography, magnetic resonance imaging, electrodiagnostic studies and local anaesthetic injections to the entrapment area. There is no definitive method to diagnose these pathologies. As a result of all these evaluations, a diagnosis can be made. There is no consensus on treatment. In isolated entrapment cases where there are no additional surgical pathologies such as space-occupying lesions, non-operative treatment is primarily recommended. It is generally recommended to try non-operative treatment for at least six months. Surgical treatment is recommended in cases where non-operative treatment fails or in cases where there are additional pathologies requiring surgery or in cases where there is extrinsic compression such as sapce-occupying lesions. In the decision and choice of surgical treatment, it is very important to determine the aetiology precisely. Surgical treatment can be performed open and arthroscopically. Various additional arthroscopic portals and techniques have been described. However, there is no clear consensus on the superiority of these treatments over each other. Although physical therapy is recommended after surgical treatment, there is no consensus on this issue in the literature.

**Aim:**

This review aims to summarize the diagnosis and management of suprascapular and axillary nerve entrapments in athletes, focusing on clinical presentation, diagnostic methods, treatment options, and current controversies.

The shoulder is a critical joint in sports for overhead motions like throwing. The repetitive movements involved in these activities subject the joint and its surrounding structures to ongoing stress and strain. Of particular significance are the neurovascular structures, which are vulnerable to injury; athletes participating in overhead sports like volleyball, tennis, and baseball frequently experience repetitive microtraumas that can damage these structures. Pathologies of neurovascular structures can often be mistaken for other common shoulder pathologies. Accurate diagnosis largely depends on the clinician’s vigilance and suspicion. Fortunately, the majority of sports-related peripheral neuropathies are classified as neurapraxia or axonotmesis, rather than complete neurotmesis [1–3]. Suprascapular and axillary nerve compressions are frequently observed in individuals participating in overhead sports or activities. The literature on these pathologies is limited, and there is no widely accepted consensus regarding their diagnosis, treatment, or management. The majority of available evidence comes from case series, with few large-scale studies to provide comprehensive insights.

## Suprascapular nerve entrapment

Suprascapular nerve pathologies are becoming widely acknowledged as a source of shoulder pain. The unique anatomy of nerve exposes it to both dynamic and static compression, as well as direct trauma [4]. The earliest studies on suprascapular nerve pathologies were conducted by Thomas in the 1930s [5]. However, the first comprehensive report on suprascapular nerve entrapment in English language was published in 1959 by Kopell and Thompson [6]. Later, in 1982, Aiello [7] identified and described nerve entrapment in the spinoglenoid region. Historically, suprascapular nerve entrapment was considered a diagnosis of exclusion. It has now become a primary diagnosis with modern diagnostic methods [8, 9].

Suprascapular nerve entrapment has been reported in over-head athletes. These include baseball players, tennis players, weightlifters, swimmers, voleyball players and kayakers [10–12]. Studies have determined that infraspinatus atrophy can be seen in the dominant shoulder, especially in this group of athletes. Since infraspinatus is frequently affected in this group and individuals are asymptomatic, it is thought that there is actually an incomplete entrapment of the nerve. It is also thought that the compression is around the spinoglenoid notch [13, 14]. However, this condition is not limited to athletes in specific sports. It can also occur in individuals engaged in overhead activities or repetitive microtrauma, such as dancers, manual labourers, cameramen or others with similar activity profiles [2, 15]. In the general population, rather than this mechanism, the most common cause of compression is a space-occupying lesion. These are mostly cysts that occur as a result of labral pathology [16].

The true incidence of suprascapular nerve entrapment is unclear. It is estimated that it accounts for around 1–2% of shoulder pain causes. However, its exact prevalence is unknown [17]. As mentioned before, it can be seen asymptomatically in overhead athletes. Therefore, it is seen as subclinical in some populations and cannot be diagnosed [2]. Among athletes, particularly volleyball, tennis and baseball players, infraspinatus atrophy has been observed in 4-34% of cases [15]. Repetitive activities and microtraumas are the main causes of nerve pathology in these athletes. Since compression is usually at the spinoglenoid notch, the infraspinatus is affected while the supraspinatus is spared. Interestingly, many of these athletes continue to perform at the same level without exhibiting any clinical symptoms. Infraspinatus atrophy in such athletes is thought to be correlate with playing duration and professional level, suggesting a link to cumulative neuropraxia [11, 15].

The suprascapular nerve was formerly known as a pure motor nerve, but is now known as a mixed nerve with a sensory component. It provides innervation to the supraspinatus and infraspinatus muscles. It also contains sensory fibers related for pain and proprioception. This sensory innervation includes structures such as the posterior shoulder capsule, glenohumeral joint, acromioclavicular joint, coracoacromial ligament, the subacromial area etc [1, 4, 9, 13, 18, 19].

The most common entrapment areas are the suprascapular and spinoglenoid notch regions. If entrapment occurs in the suprascapular notch, both the supraspinatus and infraspinatus are affected. If compression occurs in the spinoglenoid notch, the infraspinatus is affected in isolation [3, 4, 8, 13, 18, 20].

**Fig. 1:**
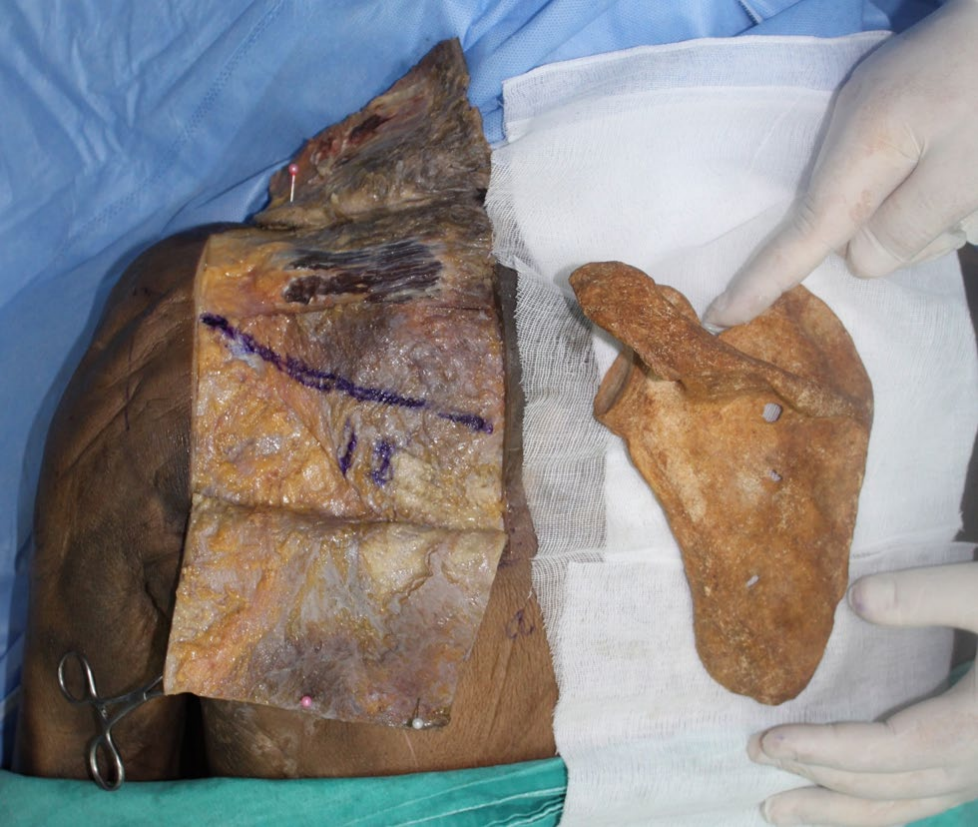
Suprascapular notch and scapular anatomy

**Fig. 2:**
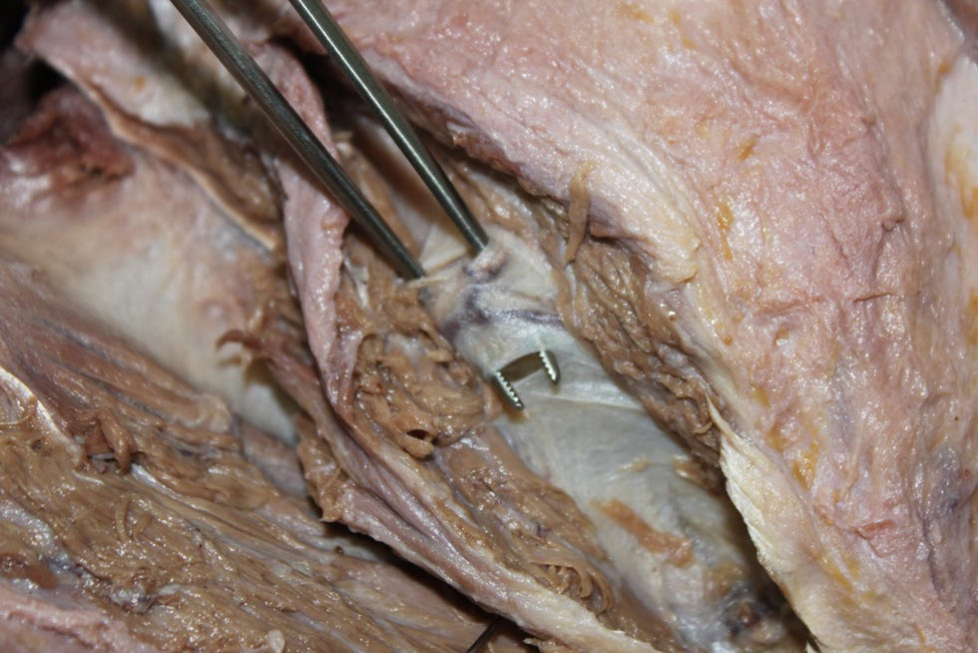
Suprascapular nerve/artery

A thorough understanding the anatomical course of the nerve is very important to determine the entrapment mechanism and localization. The suprascapular nerve arises from the upper trunk of brachial plexus at Erb’s point. It consists of C5-C6 nerve roots. In some cases, it also receives contributions from C4. Then the nerve travels through the posterior cervical triangle, parallel to the omohyoid muscle. It passes behind trapezius muscle and the clavicle before reaching the superior border of the scapula. It then passess through the suprascapular notch [1, 2, 4, 9]. The suprascapular notch is a bony structure located between origin of the coracoid process on lateral side and the transverse scapular ligament positioned superiorly. The passage of the nerve through this region is important in terms of entrapment. Various studies have examined the relationship between the anatomical structural features of this notch (size, shape, depth, etc.) and nerve entrapment **(**Fig. 1**)** [4, 21, 22]. After passing through the notch, within approximately 1 cm, it supplies motor branches to the supraspinatus muscle and receives sensory fibers from the acromioclavicular and glenohumeral joints. In this region, nerve is 3 cm away from the supraglenoid tubercle. The nerve then curves in a lateral direction toward spine of the scapula, passing the spinoglenoid notch. In some individuals, there is an anatomical structure known as the spinoglenoid ligament (also known as the inferior transverse scapular ligament) in this region. In these individuals, the nerve passes under this ligament. The nerve enters the infraspinous fossa. The nerve usually supplies two branches to innervate the supraspinatus muscle within transverse scapular ligament, usually after passing beneath it. Rarely, these branches arise before the nerve passes into the suprascapular notch, traversing over the ligament instead [1, 4, 18, 23]. Once it passes under the spinoglenoid ligament, it supplies a minimum of two branches (in some cases 3 or 4) to the infraspinatus muscle. Within the spinoglenoid notch, the nerve is at a distance of 18–23 mm from posterior rim of the glenoid [1, 9, 13, 18, 24, 25]. The spinoglenoid ligament arises from lateral portion of the scapula spine and anchors to the glenoid. It may also contain fibres that extend to the posterior joint capsule [2]. Suprascapular notch, transverse scapular ligament, and neurovascular structures vary considerably in their positional relationships to each other **(**Fig. 2**)**. The nerve typically courses beneath the ligament. The artery often runs above the ligament [1, 13]. There are also various anatomical variations of the transverse scapular ligament. Various studies suggest that hypertrophy or partial or complete ossification of this ligament may lead to compression of suprascapular nerve [4, 9, 22, 26]. In a study involving 86 cadaveric shoulders, Polguj et al. identified three types of superior transverse scapular ligaments: 54.6% were fan-shaped, 41.9% were band-shaped, and 3.5% were bifid. They also observed the anterior coracoscapular ligament, considered another potential risk factor, in 44 cadaveric shoulders [27]. Moreover, the literature highlights that the spinoglenoid ligament is also highly variable so its morphology varies, appearing as band-like, triangular or irregular [13]. Spinoglenoid ligament is a complex structure formed by the combination of both elastic and inelastic structure consisting of a dense collagen fibre bundle [28]. Studies have reported the occurence of spinoglenoid ligament in 3–100% of human cadavers [9, 29]. The spinoglenoid notch is located lateral to the spine of the scapula. Its upper border is formed by the spinoglenoid ligament mentioned earlier. The part of the spinoglenoid ligament that continues with the posterior capsule is particularly stretched during adduction and internal rotation. Upon exiting the spinoglenoid notch, nerve takes a pronounced medial turn around the base of the scapular spine. It follows along the scapular body and innervates the infraspinatus muscle [4, 18].

There are many factors in the aetiology; compressions, traction, friction, repetitive microtraumas, direct injuries, and brachial plexus pathologies. The nerve may become entrapment at any location along its course. Symptomatology varies depending on localization. In addition to the aetiological reasons just mentioned, various anatomical variations (suprascapular notch, transverse scapular ligament variations, etc.) can also contribute nerve compression. Studies have identified which a deep, narrow notch contributes to a grater predisposition to nerve compression [2, 30]. Thickening of transverse scapular ligament may also contribute to compression as previously mentioned [1]. In addition, the localization of neurovascular structures in the suprascapular notch also affects nerve entrapment. The fact that all neurological and vascular structures pass under the ligament together increases entrapment [13]. Repetitive microtraumas without any space-occupying lesion can cause nerve damage, especially in athletes who participate overhead sports. Two main mechanisms are involved in suprascapular nerve injuries: compression and traction [13]. Another mechanism is the hypothesis of nerve ischaemia due to microembolism [10]. In addition, compression mechanisms can be classified into two basic types: static and dynamic [3]. Compression resulting from repetitive microtraumas observed in overhead athletes serves as an example of dynamic compression. Similarly, traction-related injuries may occur in retracted rotator cuff tears. In addition, factors such as iatrogenic injuries, fractures, dislocations, malunions, implant irritation, and implants displaced after instability surgery are among the causes [4, 9, 13, 18]. Distinguishing between compression-related and traction-related mechanisms of suprascapular neuropathy is crucial for selecting the most appropriate treatment approach [13].

**Fig. 3:**
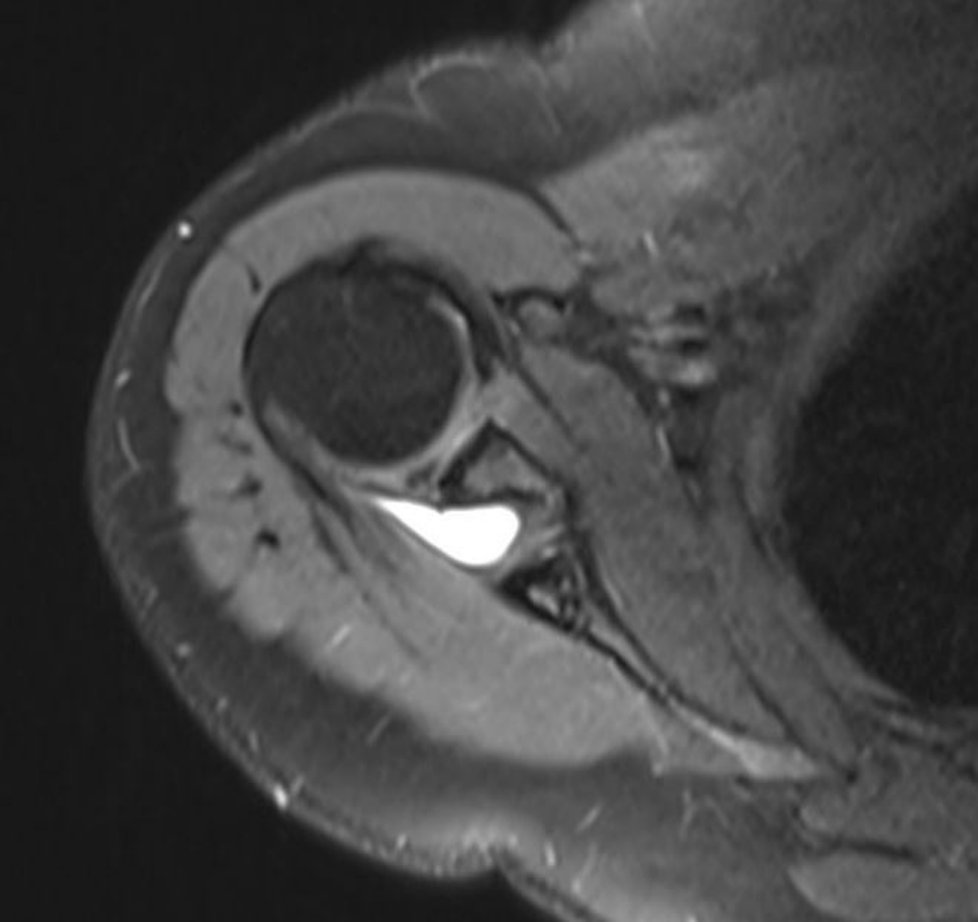
Paralabral cyst in the spinoglenoid notch

Mechanical pressure is a key factor in the mechanism of nerve compression. The duration and degree of entrapment is very important. Prolonged and severe compression often facilitates symptom recognition and diagnosis but increases the possibility of irreversible damage. On the other hand, with mild and short-term compression, diagnosis may be more challenging, but the potential for recovery is higher [31]. Extrinsic compression mechanisms are associated with space-occupying lesions, including paralabral cysts resulting from labral lesions, ganglion cysts, tumours, lipomas, and vascular malformations, spinoglenoid veins etc. **(**Fig. 3**)** [3, 10, 13, 32]. As mentioned before, anatomical variations such as ossification of transverse scapular ligament or presence of an anterior coracoscapular ligament may increase the risk [33, 34]. Transverse scapular ligament complete ossification can be observed in the literature at a rate of between 0.3% and 30%. In a study, Kim et al., ossification was detected in the transverse scapular ligament at a rate of 10.3% in patients with rotator cuff rupture [35, 36]. Paralabral cysts usually cause compression in the spinoglenoid region. Numerous studies identify these cysts as the most common cause [3, 4, 8, 37]. Traction-related damage to the suprascapular nerve can be seen in retracted rotator cuff tears. In a cadaver study, Albritton et al. demonstrated that greater retraction of the supraspinatus tendon decreased the angle between the suprascapular nerve and its first motor branch, while simultaneously increasing traction force on the nerve [38].

Dynamic compression is generally thought to be the cause of suprascapular nerve neuropathy in overhead athletes. According to a study, the prevalence of infraspinatus atrophy was reported as 12.5–34% in volleyball players, 4.4% in baseball players, and 52% in tennis players with the infraspinatus more commonly affected than supraspinatus [14]. There are several studies indicating that compression may occur in various sports and various shoulder positions. For example, suprascapular nerve is hypothesized to be compressed by upper part of infrapinatus muscle within spinoglenoid notch when the shoulder is in abduction and external rotation. Also extreme horizontal adduction, spike movements in volleyball may lead to cumulative traction-induced nerve damage. Another theory suggests that increased tension in the spinoglenoid ligament exacerbates nerve compression, particularly at the end of a throwing motion or overhead serve [4, 13]. Various studies have reported that spinoglenoid ligament tightens throughout cross-body adduction and internal rotation. This is usually seen throughout follow-through phase of throwing. Consequently, compression of the nerve may develop [2]. Additionally, repeated movements such as scapular depression, retraction, hyperabduction, and adduction combined with forward flexion are believed to contribute to nerve compression [1]. In a study conducted on elite female tennis players, an increased prevalence of clinically infraspinatus atrophy was found in the dominant shoulder. Interestingly this condition was correlated with higher performance rankings. There was no functional impairments. As long as the athlete is asymptomatic, atrophy is not thought to affect performance substantially. However, exact cause of atrophy remains unclear [15]. Another theory for suprascapular neuropathy involves intimal injury to the suprascapular or axillary artery, leading to microemboli within the vasa nervorum. It is thought that in certain instances, repetitive traction forces may cause this condition [18].

Typical clinical presentation is seen in young athletes performing overhead sports with a chronic traction-type injury etiology. This type of injury has been described in laborers who do repetitive overhead activities, such as dancers, manual labourers, cameramen or others with similar activity profiles as mentioned before [2]. In older patients with rotator cuff tears, suprascapular neuropathy should also be considered. Another scenario includes cases resulting from iatrogenic causes as well as those with a history of trauma [18].

In suprascapular nerve compression, patients usually describe a chronic, poorly localized, deep, dull ache or burning pain around posterior shoulder region. Depending on aetiology and duration of compression, pain may also occur at rest [4, 9, 10, 13] This pain may sometimes radiate to the neck and arm [4]. Some patients may experience night pain [9]. Additionally, in some cases, pain occurs or increases in intensity with overhead activities [10]. Patients sometimes describe pain as localized 3–4 cm medial to the posterolateral scapular region. Pain can also worsen during movements such as internal rotation, cross-body adduction [4].The onset of symptoms is usually insidious.

Patients may complain of weakness. In addition to a general weakness complaint, some individuals may also report of subjective weakness or fatigue during overhead movements [1, 3, 4]. Weakness and its functional limitations vary based on severity, location and duration of nerve entrapment [13, 18]. For example, compression of the spinoglenoid notch may not cause obvious weakness symptoms. The weakness that may occur in external rotation can be compensated to some extent by deltoid and teres minor muscle function. In contrast, entrapment around suprascapular notch frequently leads to functional deficits. In particular, up to 75% loss in abduction, external rotation strength can be observed [18, 35]. Suprascapular nerve compression often coexists with other pathologies, including labral tears or rotator cuff ruptures. Therefore, various symptoms may occur due to these additional pathologies. This situation sometimes makes it difficult to diagnose nerve entrapment [18].

Most critical factor for diagnosing suprascapular nerve compression in fact is clinical suspicion [4, 13]. In a systematic review [8], the primary complaint was deep posterior shoulder pain. It was reported to be seen in 97.8% of the patients. This pain may occur following overhead activities, sports, or occupational tasks (58.6%). In some cases, there is a trauma that triggers the symptoms (31%). The duration of symptoms varied widely, ranging from 0.5 to 312 months, with an average time from symptom onset to diagnosis being notably long, up to ten months in some studies [18, 35].

During clinical assessment, only shoulder should not be evaluated. The cervical spine should definitely be examined and the neurological status should be evaluated [9]. The examination should begin with inspection. The patient must be undressed and shoulders should be evaluated symmetrically. The neck region should also be evaluated in the examination. Any surgical scars or evidence of trauma should also be noted. It should be evaluated for muscle atrophy [4, 18]. Physical examination findings can vary based on duration, severity, location and aetiology. Clinical signs in early stages may be vague and minimal or difficult to detect. However, chronic compression may result more pronounced clinical findings such as muscle atrophy. In general, infraspinatus atrophy can be seen more clearly than supraspinatus since sometimes trapezius muscle can mask supraspinatus atrophy. Infraspinatus atrophy is easier to identify when the arms are positioned in forward flexion [2]. In cases of compression at suprascapular notch, both supraspinatus and infraspinatus are affected. Because of that it may cause weakness in abduction external rotation. On the other hand, compression around spinoglenoid notch primarily affects the infraspinatus muscle. As mentioned before, this weakness can be partially compensated by functions of deltoid and teres minor, and no significant weakness may be detected in this patient group [3, 4, 9, 13]. A systematic review reported visible muscle atrophy in 142 out of 182 patients (78%) and identified weakness in external rotation or abduction in 130 out of 155 patients (83.9%) [8]. However, these symptoms may not be seen in every patient group. In long-term overhead athletes, no functional deficits may be present, with painless wasting being the only sign [2, 13].

Active-passive range of motion and scapular kinematics and examination should be evaluated [18]. Tenderness is often noted over the suprascapular notch with palpation. It is located approximately 1–2 cm medial to the posterosuperior glenohumeral joint line [13]. When compression occurs in spinoglenoid notch, tenderness may be observed at the compression localization. And pain is exacerbated by cross-body adduction. Infraspinatus muscle wasting without involvement of supraspinatus, and reduced external rotation strength may present [1, 9]. Examination of muscle strength around the shoulder must be evaluated. Since it may be accompanied by other shoulder pathologies, specific examinations for other shoulder pathologies should also be performed. Additionally, careful evaluation of the cervical spine and C5-T1 nerve roots should not be overlooked [13, 18]. In the differential diagnosis, many conditions should be considered such as cervical disc pathology, Parsonage-Turner syndrome, rotator cuff disease, biceps tendon pathologies, arthritis, instability, quadrilateral space syndrome, and thoracic outlet syndrome etc [2].

**Fig. 4:**
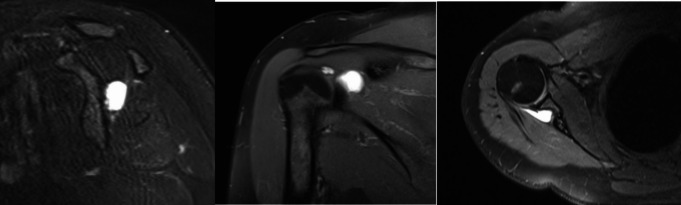
MRI images of paralabral cyst

Plain radiographs are the first-line imaging modality. Plain radiographs are useful for identifying fractures, arthritis, malunions, and other secondary bony aetiologies [1, 4, 9, 13]. However, in isolated suprascapular neuropathy, they typically do not reveal specific findings. Computed Tomography (CT) scans and 3D imaging provide better visualization and more detailed evaluations of bony structures and calcified ligaments. On the other hand they involve significant radiation exposure. Magnetic Resonance Imaging (MRI) is frequently used imaging method. MRI is the most effective tool for visualizing the nerve’s course. MRI can detect space-occupying lesions **(**Fig. 4**)**, muscle denervation findings (oedema, atrophy, and fatty infiltration etc.). Additionally, associated conditions like rotator cuff and labral pathologies can also be assessed. Ultrasound (USG) is another imaging modality cited in the literature. Electrodiagnostic studies are considered by some authors as gold standard methods. These methods are also useful for recovery progress. Electromyography (EMG) and nerve conduction velocity (NCV) studies are commonly performed to identify the exact localization of nerve entrapment [2–4, 8, 10, 13, 20]. Sensitivity and specificity vary across studies. It is important to note that EMG may be normal in early stages. If clinical findings strongly suggest compression, this should be considered a potential false-negative result [2, 4, 18].

In suprascapular nerve entrapment, local anesthetic injections can be used for diagnosis. Additionally, the localization of compression can be determined with injections. Some authors have emphasied the diagnostic accuracy of injections performed under imaging guidance [4, 13, 39]. Such injections can be used in patients with negative EMG and NCV but strong suspicion of suprascapular neuropathy. A fluoroscopy-guided injection of local anaesthetic near the suprascapular nerve can help pain relief. Injections into the suprascapular or spinoglenoid regions are typically recommended under ultrasound or fluoroscopic guidance to ensure accuracy. However, a negative response to the injection does not completely exclude the diagnosis [2, 9].

Treatment decisions should be individualized to the patient. The duration and severity of symptoms and the underlying aetiological cause must be evaluated [3, 4, 9, 10, 18]. For example, in a patient where a space-occupying lesion causes compression, surgical treatment should be prioritized over non-operative treatment. It should be determined whether the aetiological cause is dynamic or static [1, 10, 18]. Dynamic aetiologies often respond better to conservative treatment, whereas structural abnormalities may necessitate surgical intervention [4]. Some authors advocate for early intervention in cases of suprascapular nerve compression to prevent irreversible damage, although robust evidence supporting this approach is lacking. Generally, it is accepted that conservative treatment is likely to fail if a space-occupying lesion is present [9].

In the absence of a space-occupying lesion or external compression, and there is no additional pathology requiring surgical intervention, such as rotator cuff rupture, initial treatment is generally non-operatively [4, 9, 13]. Some authors suggest that symptoms can resolve with conservative management within six to 12 months [2, 18]. Non-operative treatments include activity modification, analgesics, physical therapy. Range of motion exercises are also essential to prevent secondary stiffness. Cyst aspiration is another treatment option. It is recommended that cyst aspiration be performed under imaging guidance. However it is not recommended as a first-line intervention due to limited supporting evidence. However, it may be considered for individuals not eligible for surgery or who decline surgery. Cyst aspiration should only be performed after it is certain that a cyst is present. In some cases, distended veins may be the aetiological cause. These structures can also mimic cysts [2, 8, 9, 13, 18, 20]. 

Surgical treatment is indicated in certain cases. Failure of non-operative treatment, presence of a space-occupying lesion, or the coexistence of other pathologies requiring surgical intervention can be given as an example [1, 13]. Some authors suggest nonoperative treatment may even be considered with a cyst if the duration of symptoms is short [18]. In surgical treatment, identifying the etiological factor is important. In cases of isolated nerve compression without a space-occupying lesion, decompression can be performed via either open or arthroscopic techniques. When compression is due to a paralabral cyst related to a labral pathology, surgical options include arthroscopic labral repair and cyst excision [3, 4, 8, 10]. Some studies suggest that surgery should not be postponed, arguing that waiting may render nerve damage irreversible [13]. There is currently no high-level evidence comparing surgical and conservative treatments for suprascapular nerve compression [8].

**Fig. 5:**
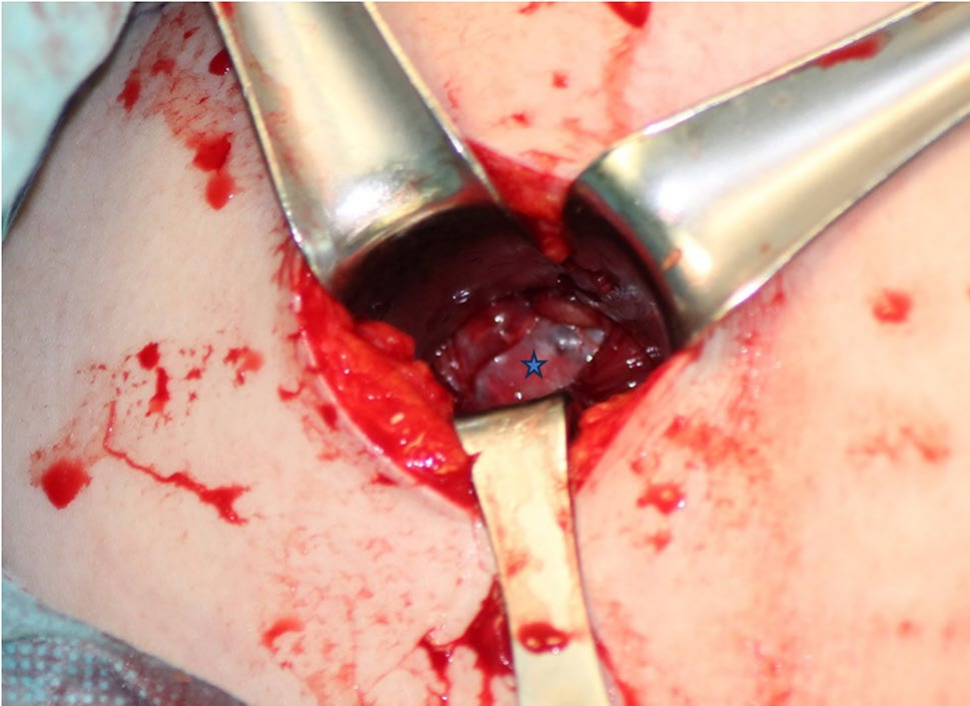
42-year-old female patient. Cyst excision was performed using mini-open surgical technique. Asterisk indicates cyst membrane

**Fig. 6:**
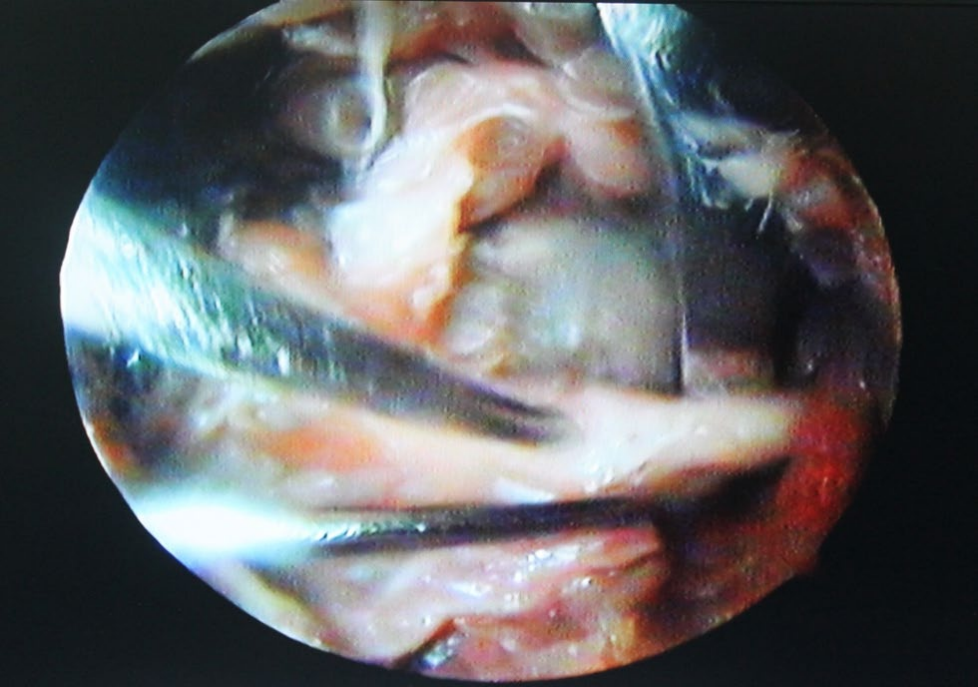
Arthroscopic release of the suprascapular nerve

Techniques involving release of transverse scapular ligament have been described for both open and arthroscopic techniques **(**Figs. 5 and 6**)**. However, there is insufficient evidence to suggest that one surgical treatment is superior to another [3, 8, 40]. The surgical approach may based on the surgeon’s experience and other patient related factors [3]. The literature suggests that open surgery should be known fundamentally. Because in some cases, such as previous surgery or deformity, arthroscopic techniques are not possible or insufficient. The traditional trapezius-splitting approach is commonly used for open surgery, although other techniques have also been described [35, 41, 42].

In cases of compression at the suprascapular notch, surgical decompression can be performed via open or arthroscopically. Surgical interventions include releasing transverse scapular ligament, deepening and widening notch (notchplasty), nerve decompression, and excision of associated cysts or other space-occupying lesions. Pain alleviation occurs in 80–96% of cases following these interventions. However, in chronic cases, muscle atrophy may not reverse. In these patients, although pain decreases after surgery, muscle atrophy may not improve [4, 9]. Arthroscopic release techniques with additional portals have been reported by Bhatia et al. [43] and Lafosse et al. [44]. In a study, Lafosse et al. reported that eight underwent postoperative EMG, with seven showing complete normalization. All patients returned to work or sports at a mean three weeks (2 days-3 months). [44].

Approaches differ in cases where rotator cuff rupture and suprascapular nerve compression occur together. Some authors advocate that isolated supraspinatus repair may be sufficient to improve neuropathy. Other authors suggest that nerve decompression should also be performed. With advancements in arthroscopy, some surgeons perform arthroscopic suprascapular release with rotator cuff repair [4, 18]. Costouros et al. [45] reported partial or complete recovery of suprascapular nerve function six months postoperatively in individuals with massive rotator cuff tears treated with arthroscopic repair. Another study found higher improvements in forward flexion and shoulder strength in patients who underwent cuff repair combined with suprascapular nerve decompression compared to those who had cuff repair alone [46].

Around spinoglenoid notch compression, a paralabral cyst or space-occupying lesion is often observed. In such cases, nonoperative treatment is usually unsuccessful. Surgical treatment should be preferred [4, 9]. Surgical options include decompression of the cyst, labral repair, or both [3, 4]. As mentioned before imaging-guided cyst aspiration may be appropriate for poor surgical candidates or patients who decline surgery. However high recurrence rates (up to 45–100%) have been reported. Literature strongly recommends labral repair, although the necessity of simultaneous cyst decompression remains debated [4, 9]. In a systematic review, it was stated that the most common labrum pathology causing compression in the spinoglenoid region was type II SLAP lesion [8]. There are different opinions about adding cyst excision to surgery. While some studies suggest that cyst excision improves clinical outcomes, others report no significant difference in results [1, 8, 47, 48]. However, the small number of participants and insufficient data make it challenging to make definitive conclusions.

Piatt et al. divided patients with suprascapular neuropathy caused by spinoglenoid cysts into four groups according to the treatment method; nonoperative treatment, needle aspiration, arthroscopic labrum-only treatment, and open or arthroscopic cyst decompression with concomitant labral fixation. The study observed higher satisfaction ratings in groups 3 and 4 compared to groups 1 and 2 [49].

In cases where there is no space-occupying lesion or cyst, dynamic etiologies are usually the cause. This is especially observed in overhead athletes. There is an overuse situation. The literature states that non-operative treatment is usually successful in such cases. Surgical treatment is indicated after at least six months of failure of nonoperative treatment. Open and arthroscopic techniques are used in surgery. There is no clear evidence yet for their superiority over each other. However, due to its minimally invasive nature and lower morbidity, arthroscopic techniques are generally recommended [4, 18]. Decompression of the spinoglenoid ligament can be performed via open or arthroscopic approaches [9, 18]. Brzoska et al. examined ten volleyball players who underwent arthroscopic decompression of the suprascapular nerve at the spinoglenoid notch. They reported improvements in shoulder function and pain, with all patients returning to competition. Range of motion increased, and partial improvement in external rotation strength was noted, although significant deficits in external rotation strength compared to the contralateral arm were observed. Recovery of muscle atrophy varied, with complete recovery in five patients (50%), partial recovery in two, and no recovery in three [16].

## Axillary nerve entrapment

The most common isolated peripheral nerve injury affecting the shoulder area in adults is the axillary nerve. Etiologies include; direct blows, dislocations, fractures, traction injuries, penetrating trauma, and quadrilateral space syndrome and as a complication due to surgery [3].

Axillary nerve compression is particularly common in young, active, and throwing athletes including volleyball players, tennis players etc [31]. Injuries especially those that occur after thoracic or shoulder surgeries can be seen a very common cause. Probably surgery is a fairly large contributor as it is the most common single nerve injury in shoulder surgery [50, 51]. One of the most common types ise compression-induced neuropraxia [52]. Neurogenic quadrilateral space syndrome (QSS) is seen 1.5 times more frequently than vascular QSS. QSS is more common in men with a ratio of 7:1. This rate is thought to be due to the male dominance in overhead sports [53].

The axillary nerve emerges from the posterior cord of the brachial plexus along with the radial nerve. It is supplied by C5-6 roots and occasionally from the C4 root. It courses lateral to the radial nerve and posterior to the axillary artery. It passes around coracoid process and then passess anterior to subscapularis muscle, and continues its course at the inferolateral border of the subscapularis, 3–5 mm medial to the musculotendinous junction. In this region, it can give sensory branches to the joint capsule. It courses very close to the inferomedial surface of joint capsule and progresses posteriorly and then reaches the quadrilateral space. The quadrilateral space is anatomically bordered by teres minor superiorly, surgical neck of the humerus laterally, long head of the triceps medially, and teres major and latissimus dorsi inferiorly. Anteriorly, subscapularis forms the boundary. In this space, axillary nerve passes superior to posterior circumflex humeral artery and divides into two branches: anterior and posterior. Anterior branch runs around surgical neck and is about 6 cm away from acromion. It innervates anterior portion of deltoid. Posterior branch has three terminal branches. These branches are sensory superolateral brachial cutaneous nerve, motor branch to the posterior portion of deltoid, and motor branch to teres minor. Middle portion of deltoid receives innervation from both anterior and posterior. The nerve also supplies sensory branches to a portion of shoulder skin and glenohumeral joint. Posterior circumflex humeral artery also follows course of axillary nerve [2, 31, 54–57]. The quadrilateral space is a region with a mean height of 2.5 cm (range: 1.3–3.3 cm), an average width of 2.5 cm (range: 1–4 cm), and an average depth of 1.5 cm (range: 1–3 cm). Axillary nerve is located in the upper lateral region of this space. It is the uppermost structure in this space [55]. As mentioned before, the proximity of axillary nerve to joint capsule and glenoid is very important. It passes near the capsule and glenoid, especially at about the 5:30 − 6 o’clock positions. This proximity should be taken into consideration during surgery for iatrogenic injuries [57].

Axillary nerve is most commonly entrapped in the quadrilateral space and known as the Quadrilateral Space Syndrome (QSS) [58]. Any situation that decreases the volume of this anatomic space can cause this syndrome. Etiologic causes include fibrous bands, repetitive strain injuries, or muscles hypertrophy. Another cause is repetitive microtraumas that occur in especially over-head athletes. Fibrous bands are considered to be the leading cause of compression. It is usually located between teres major and long head of the triceps. Studies have shown that these bands tighten during abduction - external/internal rotation. This causes the quadrilateral space volume to decrease. These bands were demonstrated in cadaver studies by McClellan and Paxinos [1, 31, 53, 56, 59]. Labral cysts, fractures, malunions, glenohumeral dislocations, haematomas, tumours, and iatrogenic injuries within this region can also damage to axillary nerve. Some humerus osteophytes may also cause nerve compression due to the close proximity of axillary nerve to the joint capsule. However, the exact pathophysiology of isolated QSS is still not clearly understood [52, 53, 55–58].

Compression mechanisms may be categorized as static or dynamic. Static causes include fibrous bands, posterior circumflex humeral artery occlusion, muscle hypertrophy, and space-occupying lesions. Dynamic causes include specific shoulder positions or positional effects of space-occupying lesions [51, 56]. Muscle hypertrophy, particularly in overhead athletes, is a frequent cause of compression [59].

QSS was first described by Cahill and Palmer in 1983 [60]. QSS can be defined as compression of the axillary nerve/posterior circumflex humeral artery within this anatomic area. Thus, QSS is considered a neurovascular syndrome. As previously discussed, many aetiologies can cause this condition. QSS predominantly affects the dominant arm in males aged 20–40 [1, 2, 59]. The condition can be classified into two subtypes: neurogenic QSS and vascular QSS. These subtypes are associated with neurological or vascular compression [53, 56, 59]. Two types of clinical conditions are frequently seen. In the first case, thrombosis occurs in posterior circumflex humeral artery after mechanical trauma. This situation is particularly observed in athletes such as volleyball players who perform repetitive abduction-external rotation. In the second case, fixed structures such as fibrous bands contribute to compression [53].

The most common symptom of axillary nerve compression is a slow-onset, poorly localized, dull aching, or burning pain in the posterior /lateral shoulder. Pain may radiate down the arm. Night pain can also occur. Symptoms typically have an insidious onset, and some patients may describe vague shoulder discomfort [1, 2, 31, 51, 53, 58]. Other symptoms are numbness, paraesthesias, fatigue, and weakness. Abduction-external rotation may aggravate symptoms. Deltoid and teres minor atrophy may be observed in chronic cases [2, 3, 59]. Deltoid fasciculations may be observed in axillary nerve compression. If there is compression of the posterior circumflex artery acute ischaemic findings, pain, pallor, diminished pulses, thrombosis, embolism, digital or hand ischemia, cold intolerance, splinter haemorrhages, cyanosis may be seen. In cases where both structures are compressed, both vascular and neurogenic findings can be seen [3, 53, 54, 59]. Cahill and Palmer stated four main findings of this pathology: diffuse pain around shoulder, paresthesias in a non-dermatomal distribution, point tenderness over the quadrilateral space, and a positive angiogram [60].

Isolated axillary nerve compression is most commonly seen in young adults under the age of 40. It is particularly seen in individuals with repetitive overhead activity. Muscle weakness in deltoid and teres minor may be observed [2, 3, 54]. Throwing athletes often describe pain during the late cocking phase of throwing. Point tenderness over the quadrilateral space is an important finding [3, 54, 59]. Two types of history may be seen in the patient history: traumatic and atraumatic. Recurrent microtraumas are often seen in non-traumatic cases. In traumatic cases, direct posterior shoulder trauma, or a history of posterior shoulder injections, iatrogenic causes may be detected [2]. Additionally, this pathology should be kept in mind in patients with persistent pain after shoulder arthroscopy [59].

Diagnosis relies on a combination of history, physical examination, and diagnostic tests [51]. Physical examination findings are often nonspecific. Tenderness over the quadrilateral space is an important finding. Compression of this area may induce pain and paraesthesias. Pain may also worsen with abduction and external rotation. Additionally, some provocative tests can be used in diagnosis. In these tests, symptoms occur when the shoulder is held in a certain position for 1–2 min. There are studies in the literature in which symptoms were provoked in forward flexion, abduction and external rotation. However, some studies indicate that this position naturally can cause compression even in asymptomatic individuals. Other studies indicate that internal rotation can also create compression, and some authors recommend performing the test with both external and internal rotation. Atrophy, in deltoid and teres minor, is observed in chronic cases. Weakness in abduction-external rotation may be observed [2, 51, 56, 59]. The differential diagnosis includes thoracic outlet syndrome, cervical spine pathologies, brachial plexus pathologies, fractures, rotator cuff tears, biceps tendon pathology, impingement, arthritis, adhesive capsulitis, instability, and suprascapular nerve injury [2, 51, 53, 54, 59].

In the study by Cahill and Palmer, angiography in abduction-external rotation is recommended, particularly in patients with cardinal QSS symptoms [60]. However, this method is not widely used today because false positives can be seen in asymptomatic individuals. No definitive diagnostic test for QSS has been identified [58, 59]. Radiological imaging can be used for diagnosis. Plain radiographs are typically the first diagnostic tool. Plain radiographs helps detect conditions such as fractures, malunions and space-occupying lesions [3, 54]. Subclavian arteriography is used to evaluate posterior circumflex humeral artery compression. However, the accuracy of vascular studies in diagnosing neurogenic QSS remains uncertain [2, 53, 54].

Nerve studies, including EMG and NCV, are generally valuable for diagnosis. However sometimes false negative results can be seen [2, 56, 58]. EMG can help identify altered nerve impulses [54]. Additionally, they can be used for follow-ups to assess recovery. However, in dynamic etiologies of QSS, EMG may not always provide accurate results [3]. However, it is still recommended for differential diagnosis [59]. Limited data are available regarding the sensitivity of EMG [53]. Teres minor is a small muscle and is very close to infraspinatus. In addition, since teres minor atrophy can be seen in this syndrome, difficulties may be experienced in needle examination. Therefore imaging methods such as USG can also be used while performing EMG to increase accuracy. However, EMG and nerve conduction studies show inconsistent results in the literature [51]. Ultrasound can also be used to identify space-occupying lesions. However, it may not be effective in cases such as the presence of fibrous bands [54]. Dynamic Doppler ultrasound may assist in identifying vascular causes [51]. MRI and CT are valuable for detecting muscle denervation, atrophy, fibrous bands, and space-occupying lesions etc [54, 56]. CT angiography may be helpful in demonstrating vascular causes. However, studies have reported that MR angiography in abduction and external rotation can yield up to 80% false-positive results in asymptomatic patients [2, 3]. In cases where QSS symptoms are absent, MRI findings of teres minor atrophy should be interpreted cautiously as nonspecific [53]. Recent studies have explored the use of 3-Tesla MR neurography in diagnosing axillary nerve compression [59].

Some authors consider the lidocaine block test to be the gold standard for diagnosing QSS. In this test, lidocaine is injected into the quadrilateral space. The test is considered positive if patient experiences immediate pain relief and can perform throwing without discomfort. However, a negative result does not rule out QSS. Because in some cases the quadrilateral space cannot be localized correctly during injection. The injection site is typically located approximately 2–3 cm inferior to the standard posterior shoulder portal. The accuracy of the procedure can be enhanced by using imaging techniques such as ultrasound [3, 51, 54, 58, 59].

The literature on QSS treatment remains limited. In the absence of penetrating injury or space-occupying lesions, non-operative treatment is recommended as the first-line treatment. Non-operative treatment is recommended for at least six months. These include rest, analgesics, activity modification, and physical therapy [2, 52–54, 59]. Physical therapy typically includes range of motion exercises, scapular stabilization exercises, strengthening of the posterior rotator cuff, stretching of the shoulder etc [2, 3, 51]. Most cases resolve with conservative management. Steroid injections are also considered one of the treatment options. But there is no strong evidence regarding their effectiveness [1, 53, 59].

Surgical treatment is indicated especially in the presence of space-occupying lesions. Another indication is failure of conservative treatment for six months. If the patient’s symptoms persist and especially if the lidocaine block test remains positive, surgical treatment is considered. The most commonly performed surgical procedure is open decompression. This procedure may include neurolysis, excision of fibrous bands, or removal of other space-occupying lesions [2, 3, 52–54]. More recently, arthroscopic techniques have been introduced as a less invasive alternative. These techniques were first described by Millett and Gaskill [61]. Arthroscopy also allows for the management of concurrent intra-articular pathologies [3, 52, 58]. In cases of vascular thrombosis, treatment options include thrombolytic medications, thrombectomy, or ligation [3, 53, 59]. Surgical outcomes for QSS are generally satisfactory, with low complication rates reported [51, 59].

Brown et al. developed an approach for QSS. If there is a suspicion of QSS, the patient’s age is first taken into consideration. If the patient is over 40 years of age, alternative diagnoses are considered. If the patient is under 40 years of age, the patient’s clinical symptoms and findings are evaluated to differentiate vascular or neurological QSS. Neurogenic QSS presents with paresthesia, weakness, fasciculations, and muscle atrophy, while vascular QSS is characterized by symptoms such as coolness, pallor, cyanosis of the hand and digits, and splinter haemorrhages. Diagnostic tools include digital subtraction angiography, computed tomography angiography, magnetic resonance angiography, ultrasound, and electromyography. In neurogenic QSS, dynamic compression occurs in the posterior circumflex humeral artery due to abduction and external rotation. In vascular QSS, a fixed occlusion due to thrombosis is observed. For neurogenic QSS initial management includes conservative therapy with analgesics and physical therapy. If conservative treatment fails, surgical intervention involving neurolysis and excision of fibrous bands or space-occupying lesions is considered. Postoperative physical therapy is recommended. A follow-up examination is recommended after three months. For vascular QSS management involves posterior humeral circumflex artery ligation with or without thrombolysis or thromboembolectomy. Anticoagulation therapy is recommended for three months, with follow-up evaluation at the end of this period [53].

There is no standardized postoperative rehabilitation protocol for QSS. Pendulum exercises and early physical therapy focusing on range of motion are recommended following surgery immediately. Some authors advise avoiding hyperextension, abduction, external rotation for four weeks postoperatively. For athletes, sport-specific therapy programs may begin as early as six weeks after surgery [59]. 

## Conclusion

Suprascapular and axillary nerve compressions are important yet often overlooked causes of shoulder pain and dysfunction, particularly in athletes engaged in repetitive overhead activities. While advancements in diagnostic methods have improved recognition of these conditions, there remains no definitive diagnostic tool or universally accepted treatment algorithm. Future research should focus on refining diagnostic criteria, optimizing surgical and non-surgical management strategies, and establishing standardized rehabilitation protocols to improve patient outcomes.

## Data Availability

No datasets were generated or analysed during the current study.
